# Supplementary material for: Adherence to the test, treat and track strategy for malaria control among prescribers, Mfantseman Municipality, Central Region, Ghana
Source: PLoS One. 2023 Feb 21;18(2):e0279712. doi: 10.1371/journal.pone.0279712 (PMC9942999; doi:10.1371/journal.pone.0279712)
Supplement: Attachment — Submitted filename: Response to Reviewers.docx [file pone.0279712.s002.docx]

Manuscript ID: PONE-D-22-22129;

Manuscript Title: Adherence to the Test, Treat and Track Strategy for Malaria Control Among Prescribers, Mfantseman Municipality, Central Region, Ghana

**POINT BY POINT RESPONSE TO COMMENTS**

**Journal Requirements:**

When submitting your revision, we need you to address these additional requirements.

1. Please ensure that your manuscript meets PLOS ONE's style requirements, including those for file naming. The PLOS ONE style templates can be found at 
<https://journals.plos.org/plosone/s/file?id=wjVg/PLOSOne_formatting_sample_main_body.pdf> and 
<https://journals.plos.org/plosone/s/file?id=ba62/PLOSOne_formatting_sample_title_authors_affiliations.pdf>

Response: **We have formatted the manuscript in line with PLOS ONE journal requirements**

2. In the ethics statement in the manuscript and in the online submission form, please provide additional information about the patient records/samples used in your retrospective study. Specifically, please ensure that you have discussed whether all data/samples were fully anonymized before you accessed them and/or whether the IRB or ethics committee waived the requirement for informed consent. If patients provided informed written consent to have data/samples from their medical records used in research, please include this information……………………..

Response: **all data set of patients were fully anonymized before we accessed the data set. This has been clearly stated in the ethics section in line 223-224**

3. We note that you have indicated that data from this study are available upon request. PLOS only allows data to be available upon request if there are legal or ethical restrictions on sharing data publicly. For more information on unacceptable data access restrictions, please see <http://journals.plos.org/plosone/s/data-availability#loc-unacceptable-data-access-restrictions>.

In your revised cover letter, please address the following prompts:

a) If there are ethical or legal restrictions on sharing a de-identified data set, please explain them in detail (e.g., data contain potentially sensitive information, data are owned by a third-party organization, etc.) and who has imposed them (e.g., an ethics committee). Please also provide contact information for a data access committee, ethics committee, or other institutional body to which data requests may be sent.

b) If there are no restrictions, please upload the minimal anonymized data set necessary to replicate your study findings as either Supporting Information files or to a stable, public repository and provide us with the relevant URLs, DOIs, or accession numbers. For a list of acceptable repositories, please see <http://journals.plos.org/plosone/s/data-availability#loc-recommended-repositories>.

We will update your Data Availability statement on your behalf to reflect the information you provide.

Response: **There are no ethical restrictions with regards to the data set used in this study, we have submitted the data set along with this manuscript**

4. Please amend the manuscript submission data (via Edit Submission) to include author Ernestina Esinam Agbemafle and Adolphina Addo-Lartey.

Response: **Thank you, we have updated the manuscript submission system.**

5. We note that Figure 1 in your submission contain [map/satellite] images which may be copyrighted. All PLOS content is published under the Creative Commons Attribution License (CC BY 4.0), which means that the manuscript, images, and Supporting Information files will be freely available online, and any third party is permitted to access, download, copy, distribute, and use these materials in any way, even commercially, with proper attribution. For these reasons, we cannot publish previously copyrighted maps or satellite images created using proprietary data, such as Google software (Google Maps, Street View, and Earth). For more information, see our copyright guidelines: <http://journals.plos.org/plosone/s/licenses-and-copyright>.
 
We require you to either (a) present written permission from the copyright holder to publish these figures specifically under the CC BY 4.0 license, or (b) remove the figures from your submission:
 
a. You may seek permission from the original copyright holder of Figure 1 to publish the content specifically under the CC BY 4.0 license.  
 
We recommend that you contact the original copyright holder with the Content Permission Form (<http://journals.plos.org/plosone/s/file?id=7c09/content-permission-form.pdf>) and the following text:
“I request permission for the open-access journal PLOS ONE to publish XXX under the Creative Commons Attribution License (CCAL) CC BY 4.0 (<http://creativecommons.org/licenses/by/4.0/>). Please be aware that this license allows unrestricted use and distribution, even commercially, by third parties. Please reply and provide explicit written permission to publish XXX under a CC BY license and complete the attached form.”
 
Please upload the completed Content Permission Form or other proof of granted permissions as an "Other" file with your submission.
 
In the figure caption of the copyrighted figure, please include the following text: “Reprinted from [ref] under a CC BY license, with permission from [name of publisher], original copyright [original copyright year].”
 
b. If you are unable to obtain permission from the original copyright holder to publish these figures under the CC BY 4.0 license or if the copyright holder’s requirements are incompatible with the CC BY 4.0 license, please either i) remove the figure or ii) supply a replacement figure that complies with the CC BY 4.0 license. Please check copyright information on all replacement figures and update the figure caption with source information. If applicable, please specify in the figure caption text when a figure is similar but not identical to the original image and is therefore for illustrative purposes only.
The following resources for replacing copyrighted map figures may be helpful:
 
USGS National Map Viewer (public domain): <http://viewer.nationalmap.gov/viewer/>
The Gateway to Astronaut Photography of Earth (public domain): <http://eol.jsc.nasa.gov/sseop/clickmap/>
Maps at the CIA (public domain): <https://www.cia.gov/library/publications/the-world-factbook/index.html> and <https://www.cia.gov/library/publications/cia-maps-publications/index.html>
NASA Earth Observatory (public domain): <http://earthobservatory.nasa.gov/>
Landsat: <http://landsat.visibleearth.nasa.gov/>
USGS EROS (Earth Resources Observatory and Science (EROS) Center) (public domain): [http://eros.usgs.gov/#](http://eros.usgs.gov/)
Natural Earth (public domain): <http://www.naturalearthdata.com/>

Response: **Figure 1 was drawn by authors using the Quantum Geographical information System (QGIS) and has no issues relating to copywrite. However, we have described the study site extensively and deleted the figure completely from the manuscript.**

6. We note you have included a table to which you do not refer in the text of your manuscript. Please ensure that you refer to Table 2 in your text; if accepted, production will need this reference to link the reader to the Table.

Response: **Table 2 has been appropriately referred to in line 278-289**

**Reviewers' comments**

**Comments to the Author**

Reviewer#1:

**Introduction:**
1. The main aim of the study is “to assess the level of adherence to the T3 strategy”. It appears the assessment has been done at two distinct levels (1) Identify the outcome (by review of patient records) (2) Identify the exposure variables (an important component is interview of the prescribers). Since the methods of assessment of these two are completely different, they may be mentioned separately. For example, there is confusion about study population and study subjects (the study subjects should come from the study population).

Response: **The two distinct methods used in the assessment have been described under study design as suggested and the corrections have been made regarding study population and study subjects (lines 111-115)**

Methods:
2. Study population (lines 121-127): A brief description about the health care system (where does the patient go first? Is it free or on payment? Who is authorized to treat? What is the routine method for diagnosis of malaria? Who were the prescribers? How follow up is enforced? Etc). Some background information regarding educational background and health care responsibility of medical officers, physician assistants, and nurse prescribers may be mentioned.

Response: **A brief description of the healthcare system and background information about prescribers have been provided under the operation of the health care system (lines 136-158)**

3. Sample Size: The necessity of sampling will be realized only when the total number of records available for analysis is known. It will be easier for the readers to understand if the values of each component of the formula for sample size calculation is mentioned (e.g, there is no mention of the confidence level.)

Response: **The total number of record available for analysis was earlier mentioned under sampling, however, for clarity, sampling and sample size calculation has been merged to provide a good flow of information (lines 163-180). Also, the terms in the formula for sample size calculation have been described and 95% confidence interval specified (160-181)**

4. Sampling: What was the justification of using systematic random sampling instead of simple random sampling? What was the interval?

Response: **Thank you for pointing this out. Due to the large number of datasets, we used systematic sampling to ensure that the data selected is spread across the patients seen at all hours of each day since facilities provide 24/7 care and the electronic database captures as such. Sampling interval was calculated for each site based on their average daily number of cases and used (interval of 2 for both sites). This has been explained further in the write-up (Lines175-185)**

5. Outcome: Each individual component of the outcome (T3) can be treated as independent, because they are influenced by different predictors. For example testing will depend on the availability of the RDT and laboratory facilities, while treating will depend on availability of antimalarial drugs. Therefore, individual components of T3 can be treated as outcome and appropriate exposure variables may be selected accordingly.

Response: **Thank you for this observation.** **it is very true that each of the component of the outcome (T3) can be treated as independent, however, authors did not consider using each of the components as an individual outcome and did not determine the corresponding exposure variable. Ours was a retrospective study; it will be very easy to take this into consideration when conducting a prospective study. We have therefore recommended this to be considered in future studies and also added it to the limitations of the study (lines 430-432 and 456-458).**

6. Exposure variables: Since the prescribers belong to three different categories (e.g, medical officers, physician assistants, and nurse prescribers), whose professional competence may vary widely, the influence of these categories on the outcome can be studied separately. Since, predictor variables have not been defined clearly, all sorts of associations have been tried.

Response **it is very true that prescribers belong to different categories whose competencies vary widely, however the authors decided to combine these professionals because the study was conducted only in the Mfantseman municipality and considering only particular groups will result in smaller sample size. Moreso, training of prescribers on the T3 strategy is a common variable that is likely to influence adherence no matter the professional category of the prescriber. We instead considered the categories as a factor that may be associated with adherence to the T3 strategy ( Table 2)**

Discussion:
7. Discussion has concentrated more on the comparison with other studies but less on the possible causes of failure.

Response: **Thank you for this observation. We have described possible causes of failure; examples include mentioning of the possibility of non-adherence to the testing component of the strategy in the hospitals being the longer waiting time associated with receiving laboratory results. The longer wait period might have contributed to the lower levels of testing in urban settings and also facilities that offer microscopy testing service. (Lines 314-423)**

***With regards to the treatment patterns, authors attributed this to the availability of the recommended antimalarials in health facilities; prescribers are more likely to prescribe treatment based on the availability of ACTs in the health facility (line 319-323)***

Reviewer 2

Comments
1. The introduction section should highlight the importance of the study from programme point of view.

Response: **This has been addressed in the introduction section (lines 68-86)**

2. The authors should check the discussion section because many from line no 258 to 310 missing and 310 -326 wrongly uploaded.

**Response: Thank you, all missing sentences in this section have been inserted (lines 296-423)**

Reviewer 3

1. The introduction section has not clearly mentioned the objective/importance of the present study

Response: **the objectives of the study have been clearly mentioned in the introduction (Lines 106 to 108)**

2. The method section needs improvement. The authors should have mentioned only the essential and critical steps in different sub headings

**Response: this is well noted. We have revised and mentioned critical steps to ensure good flow of information in the methods section (lines 110-231)**

3. In the result section the manufacturer of RDTs used for diagnosis and the partner drug of ACT administered should be clearly mentioned.

Response: **Thank you for this suggestion. However, authors maintained the generic names of the drugs and used RDT’s in general because various ACTs are administered in health facilities in Ghana. Since our focus was not on the efficacy of the drug or the RDT’s but on whether the T3 strategy was adhered to, we chose to omit this information for the drugs and RDT’s as it was not collected as part of our retrospective study.**

4.The caption of Table 2 should be precise.

Response: **The table heading has been precisely labelled (lines 284 -285)**

5. The discussion section is missing many of the sentences from line 258- 326.

Response: **Thank you, all missing sentences in this section have been inserted and modified from (line 299-426)**
